# Antenatal Glucocorticoid Treatment Affects Hippocampal Development in Mice

**DOI:** 10.1371/journal.pone.0085671

**Published:** 2014-01-22

**Authors:** Cornelle W. Noorlander, Deodata Tijsseling, Ellen V. S. Hessel, Willem B. de Vries, Jan B. Derks, Gerard H. A. Visser, Pierre N. E. de Graan

**Affiliations:** 1 Brain Center Rudolf Magnus, Department of Neuroscience and Pharmacology, University Medical Center Utrecht, Utrecht, The Netherlands; 2 Department of Obstetrics, University Medical Center Utrecht, Utrecht, The Netherlands; 3 Department of Neonatology, University Medical Center Utrecht, Utrecht, The Netherlands; Washington University, School of Medicine, United States of America

## Abstract

Synthetic glucocorticoids are administered to pregnant women at risk for preterm delivery, to enhance fetal lung maturation. The benefit of this treatment is well established, however caution is necessary because of possible unwanted side effects on development of different organ systems, including the brain. Actions of glucocorticoids are mediated by corticosteroid receptors, which are highly expressed in the hippocampus, a brain structure involved in cognitive functions. Therefore, we analyzed the effects of a single antenatal dexamethasone treatment on the development of the mouse hippocampus. A clinically relevant dose of dexamethasone (0.4 mg/kg) was administered to pregnant mice at embryonic day 15.5 and the hippocampus was analyzed from embryonic day 16 until adulthood. We investigated the effects of dexamethasone treatment on anatomical changes, apoptosis and proliferation in the hippocampus, hippocampal volume and on total body weight. Our results show that dexamethasone treatment reduced body weight and hippocampal volume transiently during development, but these effects were no longer detected at adulthood. Dexamethasone treatment increased the number of apoptotic cells in the hippocampus until birth, but postnatally no effects of dexamethasone treatment on apoptosis were found. During the phase with increased apoptosis, dexamethasone treatment reduced the number of proliferating cells in the subgranular zone of the dentate gyrus. The number of proliferative cells was increased at postnatal day 5 and 10, but was decreased again at the adult stage. This latter long-term and negative effect of antenatal dexamethasone treatment on the number of proliferative cells in the hippocampus may have important implications for hippocampal network function.

## Introduction

Pregnant women at risk for preterm delivery are treated with high doses of synthetic glucocorticoids (GCs), such as dexamethasone (dex) or betamethasone, to enhance fetal lung maturation. Although this treatment is highly effective in reducing morbidity and mortality of the preterm neonate [1], increasing information is available about the adverse side effects of GC treatment. GC treatment directly influences the development of the fetus after crossing the placenta and entering the fetal circulation [2]. The action of GCs is mediated by its interaction with the glucocorticoid receptor and/or the mineralocorticoid receptor, which are abundantly expressed in the hippocampus [3], [4]. The hippocampus, an important brain center involved in cognitive functions, can be affected by elevated levels of GCs, by influencing cell death and proliferation. Coe and co-workers [5] have shown that prenatal stress, causing elevated GCs levels, diminishes neurogenesis in the dentate gyrus (DG) of juvenile rhesus monkeys and that GCs may suppress cell proliferation. Moreover, acute administration of dex in rats results in neuronal death of granule cells in the DG, and pyramidal neurons in the cornu ammonal (CA) subfields of the hippocampus [6]–[8]. In addition, chronically elevated GCs damage hippocampal pyramidal neurons in the CA and inhibit neurogenesis in the adult rat DG [9]–[12]. Granule cells in the DG are capable of proliferating throughout adulthood by neurogenesis from progenitors located in the subgranular zone of the DG [13], [14]. Many studies indicate a relationship between death and birth of neurons and suggest that neurogenesis does occur to maintain neuron numbers, especially after injury [15]–[18].

Although many studies have focused on GC-induced damage to the hippocampus, little is known about the effects of GCs throughout hippocampal development. To investigate the development of the hippocampus after a single antenatal dex treatment in mice, we used a treatment protocol resembling that used in the human clinical situation and studied apoptosis, cell proliferation and hippocampal volume during prenatal and postnatal life and adulthood.

## Materials and Methods

### Animals

Pregnant mice C57Bl/6-JIco (Charles River Laboratory, France) were housed individually on day eight of pregnancy. Pregnancy was determined by observation of a vaginal plug. Following timed exposure to the male, the plug date was considered day 0 of gestation. On day 15.5 of pregnancy, the mice were injected intraperitoneally with either dexamethasone (0.4 mg/kg, Dexamethasone Sodium Phosphate; BUFA, The Netherlands) or with equal volumes of sterile saline. Women threatening to deliver preterm are often administered 6 mg dex four times within 48 hours. With an average weight of around 75 kg, this results in 4 times 0.08 mg/kg dex with a plasma half-life of 3 hours in the human. Plasma half life of dex in mice is unknown; we therefore decided to give one injection of an equivalent dose of dex 0.4 mg/kg. The comparison of the stage of brain development, is a major concern with regard to the interpretation of animal studies looking at the effects of GCs on neural measures [19]. Estimates of the mouse equivalent age of a term human in respect of neural development have ranged from 5.5 to 19 days of postnatal age [20], [21] with a general consensus that a 8- to 11-day-old mouse is equivalent to a term human fetus in terms of brain development. Term mice are therefore best comparable to preterm human fetuses, exactly those who receive prenatal GCs *in utero*. When referring to the administration of dexamethasone in this paper, it should be noted that we have used dexamethasone sodium phosphate like in the clinical situation, which has a larger molecular weight than dexamethasone (516.4 versus 392.5). As a result, a 0.4 mg/kg injection of dexamethasone sodium phosphate is equivalent to a 0.3 mg/kg dose of dexamethasone.

Mice were allowed ad libitum access to food and water. Light/dark cycle (dark phase 1900-0700 h), temperature (21°C) and humidity (60%) were kept constant. Pups were sacrificed by decapitation and studied at seven different time-points; at embryonic day (E) 16, E18, postnatal day (P) 0, P5, P10, P20 and at the age of 6 months. For the adult stage, pups were weaned at P25 and remained group-housed two to four per cage with same-sex littermates until they were sacrificed and studied at 6 months of age (adult). Sixteen randomly chosen pups were sacrificed per time point (dex n = 8, sal n = 8). Only the heaviest and smallest pup from anyone litter were not included in the experiments. Males and females were equally distributed among the groups. All experimental procedures were approved by the Committee for Animal Experimentation of the University of Utrecht.

### Tissue processing

Embryos or brains of mice were dissected and immediately fixed overnight in 4% paraformaldehyde in 0.1 M phosphate buffer, pH 7.4, at 4°C. After fixation, samples were dehydrated and embedded in paraffin. The entire hippocampus was sliced in 7 µm thick coronal sections and mounted on SuperFrost plus slides (Menzel Gläser, Germany). All subsequent quantitative analyses were performed with the observer blind to the treatment group.

### Nissl-staining

Serial sections obtained from mice at different developmental stages were deparaffinated, rinsed in water, stained for 10 minutes in 0.5% Cresyl Violet and briefly rinsed in an acetate buffer, pH 4. The sections were then differentiated in 96% ethanol for 30 seconds, dehydrated in 100% ethanol, cleared in xylene and mounted with Entellan.

### Immunohistochemistry

Serial sections obtained from mice at different developmental stages were deparaffinated, rinsed in water, submitted to microwave treatment (7 min. 650 W and 5 min. 350 W) in 0.01 M Sodium Citrate buffer (pH 6), and incubated in 0.3% H_2_O_2_ in tris-buffered saline (TBS) for 30 min to reduce endogenous peroxidase activity. Then, sections were washed in TBS, blocked with 4% fetal calf serum in TBS for 30 min and incubated overnight at room temperature with rabbit anti-Ki67 (Chemicon International Inc, USA; 1: 500), or rabbit anti-active caspase-3 (Biovision, USA; 1∶100) in TBS. The next day, sections were washed three times with TBS for 5 min, incubated for 1 h with biotinylated goat anti-rabbit immunoglobulin in TBS (1∶1000), washed three times with TBS for 5 min, incubated for 1 h with avidin-biotin-peroxidase reagents (ABC elite kit, Vector Laboratories, UK; 1: 1000) in TBS and washed with TBS three times for 5 min. The slides were stained with DAB (3,3′-diamino-benzidine), were washed twice with demineralized water for 5 min, dehydrated with ethanol and mounted using Entellan.

### Quantification and stereology

Nissl-stained serial sections (7 µm) were used for stereological quantification and measurements were performed using an image based analysis system. Neuronal density and volume were calculated in the CA pyramidal cell layer and in the granule cell layer of the dentate gyrus (DG), using the optical dissector method [22]. Object-Image software was used to randomly place a square counting frame over the cell layer on the section. Individual sections were viewed on a video monitor connected to a Zeiss microscope at a final magnification of 40× and counted if they were positioned within the counting frame or intersected by its inclusion edges. The total number of neurons was calculated from the neuronal density and the total volume of the cell layer. Quantification of Ki-67-immunoreactive cells was performed in hippocampal sections (coronal) adjacent to those stained with cresyl violet in the subgranular zone of the DG. The subgranular zone was defined as a two-cell layer thick zone along the inner border of the granule cell layer. Caspase-3 positive cells were counted in the pyramidal cell layer of the CA and in the granule cell layer of the DG.

### Statistical analysis

Statistical analysis was performed using Two-Way ANOVA followed by the Bonferroni *post hoc* test. Data are presented as means ± SD. P values <0.05 were accepted as statistically significant.

## Results

### Body weight and hippocampal volume

**Figure 1:**
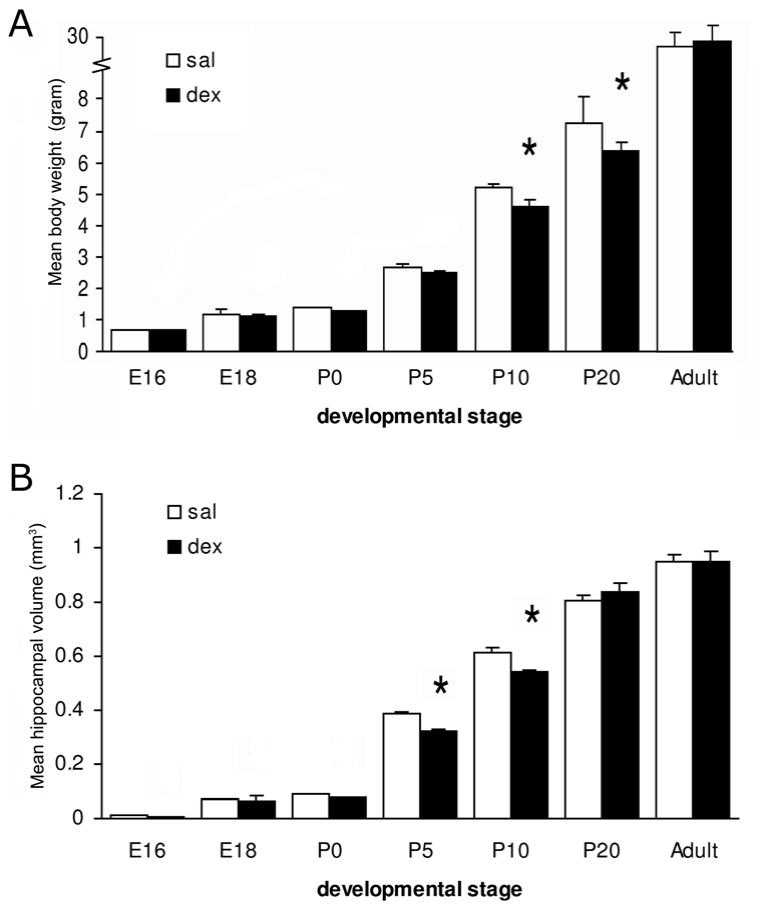
Effect of antenatal dexamethasone treatment on body weight and hippocampal volume. Data are presented as mean ± SD. Dex-treated mice showed a significant reduction in mean body weight at P10 and P20 and reduced hippocampal volume at P5 and P10. E: embryonic day, P: postnatal day, sal: saline-treatment (n = 8), dex: dexamethasone-treatment (n = 8), * p<0.05 (Two-Way ANOVA+Bonferroni).

Figure 1 shows the effects of a single antenatal dex treatment on fetal, neonatal and adult body weight (Fig. 1A) and hippocampal volume (Fig. 1B). The dex group showed a significantly lower body weight as compared to the saline group at P10 (5.19±0.09 g in sal-treated vs. 4.68±0.13 g in dex treated animals; P<0.01) and P20 (sal: 7.35±0.70 g vs. dex: 6.42±0.28 g; P<0.0001). Interaction treatment x time F(1,98) = 7.569, P<0.0001; treatment F(1,98) = 22820, P<0.0001; time (F1,98) = 19.60, P<0.0001. At E16, E18, P0, P5 and adult stage body weights were similar between both groups. Furthermore, after dex treatment a reduction in total hippocampal volume was observed at P5 (sal: 0.386±0.013 mm^3^ vs. dex: 0.324±0.018 mm^3^; P<0.01) and P10 (sal: 0.615±0.041 mm^3^ vs. dex: 0.539±0.027 mm^3^; P<0.001). Interaction treatment x time F(1,98) = 4.217, P = 0.0008; treatment F(1,98) = 1686, P<0.0001; time F(1,98) = 5.965, P = 0.0164. At E16, E18, P0, P20 and adult stage, dex treated animals did not differ in hippocampal volume compared to the saline group.

### Volume and cell number

**Figure 2:**
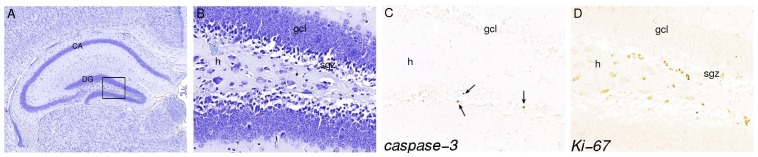
Typical example of a hippocampus of P20, stained with Nissl (A, B), immunohistochemistry for active-caspase-3 (C) and Ki-67 (D). Panel B, C and D represent a magnification (40×) of the boxed area in panel A (10×). Arrows in panel C show apoptotic cells in gcl. Proliferative cells are detected in the sgz of the DG. h = hilus, gcl = granule cell layer, sgz = subgranular zone.

**Figure 3:**
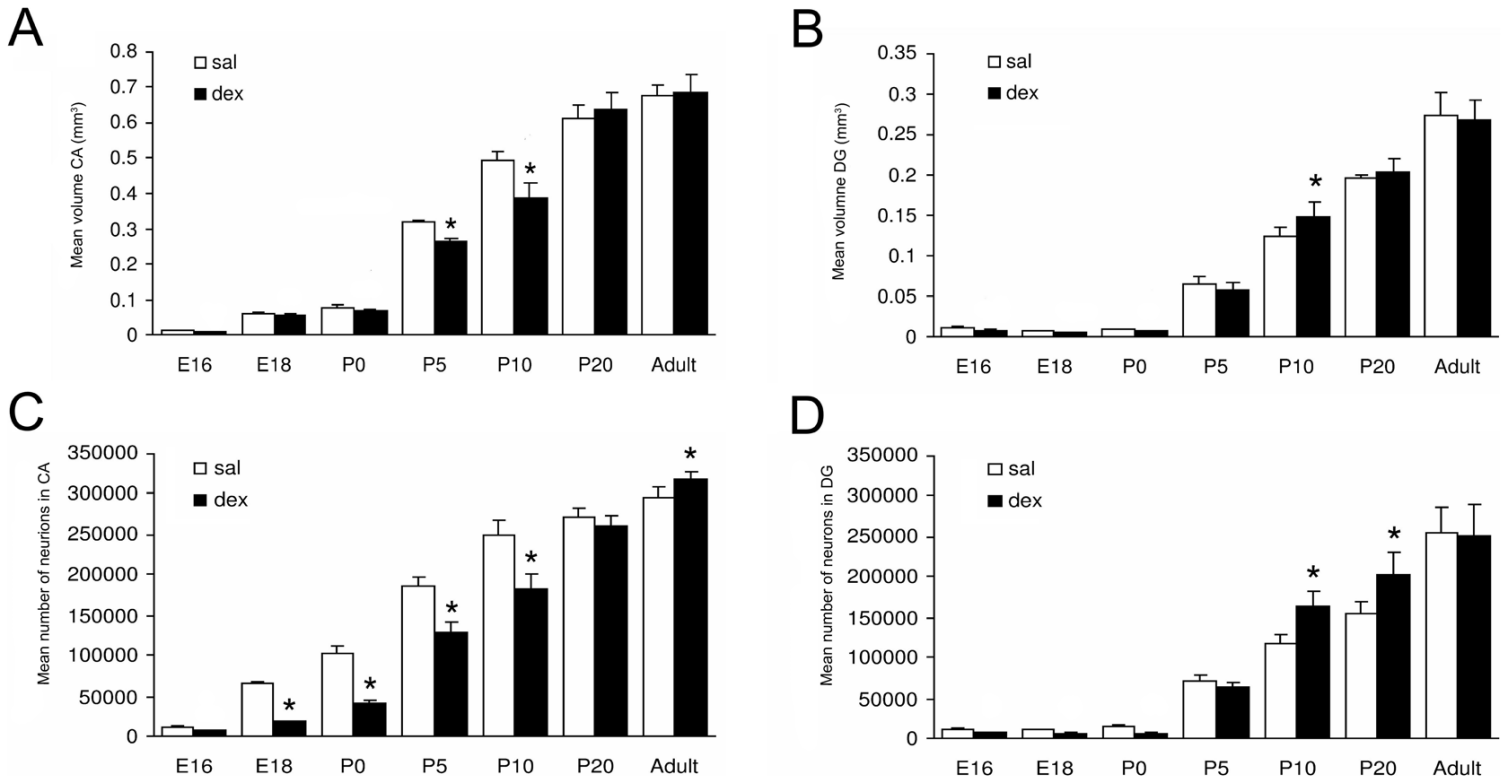
Effect of antenatal dexamethasone treatment on the volume (A, B) and total number of neurons (C, D) of the hippocampus. Data are presented as mean ± SD. Panel A and C show a significant decrease in volume of the CA at P5 and P10 and in total number of neurons in the CA area at E18 until P10 and an increase of number of neurons in the CA of the dex-treated group at adulthood. Panel B shows an increase in volume of the DG of the dex-treated group at P10. Panel D shows a significant increase in total number of neurons in the DG of the dex-treated group at P10 and P20. Sal: saline-treatment (n = 8), dex: dexamethasone-treatment (n = 8). * p<0.05 (Two-Way ANOVA+Bonferroni).

To investigate the effects of dex on the hippocampus, Nissl-stained coronal sections were used (Fig. 2A,B). Examination of the hippocampus did not reveal any disturbances in cellular morphology or anatomical organization after antenatal dex treatment at any of the developmental stages analyzed. The hippocampus was subdivided into CA and DG subregions and volume and total cell numbers were determined in hippocampus sections from E16 till adult stage. Figure 3 shows that the volume and total number of neurons in the CA and DG increased during hippocampal development in both the sal and the dex group. In the CA, a significantly lower volume was found after dex treatment, at P5 (sal: 0.321±0.004 mm^3^ vs. dex: 0.265±0.009 mm^3^; P<0.001) and P10 (sal: 0.491±0.029 mm^3^ vs. dex: 0.391±0.037 mm^3^; P<0.0001) (Fig. 3A). Interaction treatment x time F(1,98) = 10.16, P<0.0001; treatment F(1,98) = 1673, P<0.0001; time F(1,98) = 15.13, P = 0.0002. No difference in volume of the CA was detected between the sal and the dex group at E16, E18, P0, P20 and adult stage. However, when we studied the total number of neurons in the CA, we found a significantly lower total number of neurons in the CA at E18 (sal: 65631±2516 vs. dex: 18523±959; P<0.0001) until P10 (P0 sal: 102000±9525 vs. dex: 42300±2622; P5 sal: 185521±11215 vs. dex: 128520±13205; P10 sal: 249000±18288 vs. dex: 182000±17888; all P<0.0001) in the dex treated animals. At adult stage a significantly increased number of neurons was found in these animals (sal: 296000±12297 vs. dex: 318000±9357; P<0.001). Interaction treatment x time F(1,98) = 41.16, P<0.0001; treatment F(1,98) = 1880, P<0.0001; time F(1,98) = 251.9, P<0.0001. The two groups did not differ in total number of neurons in the CA at E16 and P20 (Fig. 3C). The DG of the hippocampus had a significantly increased volume after dex treatment compared to the saline group at P10 (sal: 0.124±0.012 mm^3^ vs. dex: 0.148±0.020 mm^3^; P<0.01). Interaction treatment x time F(1,98) = 2.416, P = 0.0321; treatment F(1,98) = 967.9, P<0.0001; time NS (not significant, F<1) (Fig. 3B). No difference in volume of the DG was found between the groups, from E16 until P5, at P20 and adult stage. A significantly higher number of neurons in the DG was observed in the dex group at P10 (sal: 117000±11959 vs. dex: 164000±17786; P<0.0001) and P20 (sal: 155255±14717.91 vs. dex: 203194±28303; P<0.0001) (Fig. 3D). At E16 until P5 and at adult stage the groups did not differ in number of neurons in the DG. Interaction treatment x time F(1,98) = 9.779, P<0.0001; treatment F(1,98) = 540.6, P<0.0001; time F(1,98) = 8.564, P = 0.0043.

### Apoptosis

**Figure 4:**
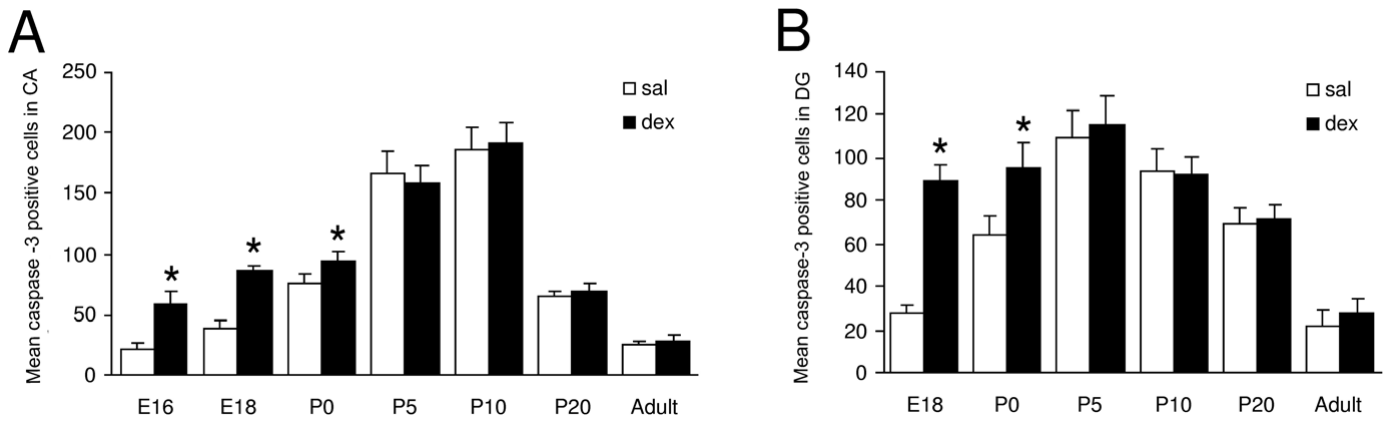
Effect of antenatal dexamethasone treatment on the total number of apoptotic cells in the pyramidal cell layer in the CA (A) and in the granule cell layer of the DG (B). Data are presented as mean ± SD. Apoptosis is significantly increased in the dex-treated group in both the CA area (E16, E18 and P0) and the DG (E18 and P0). Sal: saline-treatment (n = 8), dex: dexamethasone-treatment (n = 8), * p<0.05 (Two-Way ANOVA+Bonferroni).

To investigate the effects of antenatal dex treatment on the number of apoptotic cells in the CA and DG, active-caspase-3 positive cells were counted at different stages during hippocampal development (Fig. 2C). Analysis of the number of apoptotic cells started at E18, since in the mouse hippocampus the CA is not distinguishable from the DG at earlier stages of development. In both experimental groups, the total number of apoptotic cells in the CA increased during development until P10, and decreased thereafter (Fig. 4A). The number of apoptotic cells in the DG also increased during development, but already after P5 the number of apoptotic cells decreased (Fig. 4B). Significantly more apoptotic cells were detected after dex treatment in both the CA and DG. In the CA at E16 (sal: 21±5.6 vs. dex: 59±8.9; P<0.0001), E18 (sal: 39±6.5 vs. dex: 86±4.2; P<0.0001) and P0 (sal: 75±8.2 vs. dex: 94±7.6; P<0.01) and in the DG at E18 (sal: 28±3.5 vs. dex: 89±7.6; P<0.0001) and P0 (sal: 64±8.3 vs. dex: 95±11.6; P<0.0001). No differences in the number of apoptotic cells were detected postnatally (P5, P10, P20 and adult stage) in either the CA or DG area of the hippocampus between the experimental groups (Fig. 4). Interaction treatment x time active caspase-3 in the CA F(1,98) = 14.86, P<0.0001; treatment F(1,98) = 555.4, P<0.0001; time F(1,98) = 60.35, P<0.0001. Interaction treatment x time active caspase-3 in the DG: F(1,84) = 28.34, P<0.0001; treatment F(1,84) = 171.8, P<0.0001; time F(1,84) = 86.30, P<0.0001.

### Proliferation

**Figure 5:**
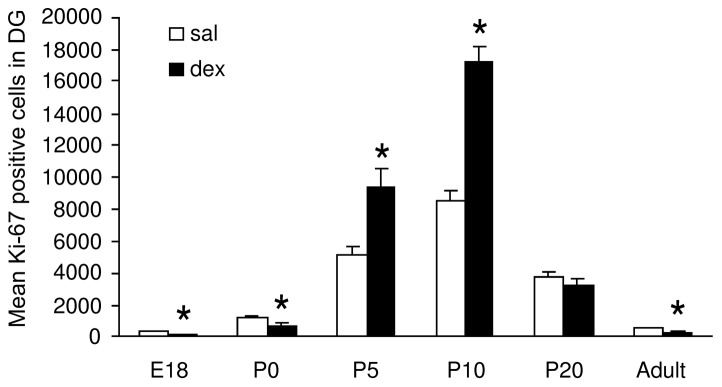
Effect of antenatal dexamethasone treatment on the total number of proliferative cells in the subgranular zone of the DG. Data are presented as mean ± SD. At E18 and P0, the number of proliferative cells is decreased in the dex-treated group, while an increase is observed at P5 and P10. At the adult stage the number of proliferative cells are decreased in the dex-treated group. Sal: saline-treatment (n = 8), dex: dexamethasone-treatment (n = 8), * p<0.05 (Two-Way ANOVA+Bonferroni).

The effects of dex on proliferation were visualized using an antibody against Ki-67 (Fig. 2D), a nuclear antigen that is expressed during all stages of the cell cycle except G0 [23], [24]. During hippocampal development and in the adult hippocampus, significant numbers of proliferating cells were detected mainly in the subgranular zone (SGZ) along both blades of the dentate gyrus, occasionally in the hilus and only rarely in the granule cell layer (Fig. 2D). Analysis of the number of proliferating cells in the SGZ started at E18, because in the mouse hippocampus the CA is not distinguishable from the DG at earlier stages of development. Immunohistochemical analysis of Ki-67 showed that antenatal dex treatment was associated with a lower number of proliferating cells in the SGZ of the DG compared to the saline-treated group at E18 (sal: 349±29 vs. dex: 125±18; P<0.0001) and P0 (sal: 1148±156 vs. dex: 659±184; P<0.0001) (Fig. 5). However, at P5 and P10, the number of proliferating cells was higher in the dex than in the saline group (P5 sal: 5064±584 vs. dex: 9453±1058; P10 sal: 8502±651 vs. dex: 17182±954; both P<0.0001). At P20 no difference was observed while at adult stage the number of proliferating cells was again significantly lower after antenatal dex treatment (sal: 510±60 vs. dex: 230±46; P<0.0001). Proliferation was analyzed as percentage of control (100%). Interaction treatment x time F(1,84) = 46.42, P<0.0001; treatment F(1,84) = 46.2, P<0.0001; time NS (F<1).

## Discussion

In the present study, we have focused on the effects of a single antenatal dex treatment on the development of the mouse hippocampus. By giving 0.4 mg/kg dex to pregnant mice we have attempted to replicate the human situation of a single course of antenatal GCs. Shortly after dex treatment apoptosis was increased in both the CA and DG and proliferation was reduced in the SGZ of the DG of the fetal hippocampus. This was followed by enhanced proliferation postnatally, but at adulthood, the number of proliferative cells was lower than in the control group. Body weight, hippocampal volume and the total number of neurons in the CA and DG were reduced by dex administration, but these effects were transient and did not persist in adulthood.

We showed that dex treatment decreased the total number of neurons in the CA at E18 until P10 and the volume of the CA at P5 and P10. This decrease is probably caused by increased apoptosis as shown by the large number of apoptotic cells detected directly after the treatment, at E16, E18 and P0. This finding in the CA area of the hippocampus is in agreement with the findings by Haynes et al. [7], who reported dramatic neuronal cell death in the CA area of rats, with the CA1 and CA3 subfields being particularly vulnerable for acute dex treatment. However, they administered a high dose of dex directly in the rat, while we administered a lower dose prenatally to the mother (20 mg/kg and 0.4 mg/kg, respectively). In an observational study in newborn infants who died within 4 days after delivery, we also found a decreased neuronal density in the CA area of the hippocampus of neonates treated antenatally with GCs [25]. Chronic stress and long-term administration of GCs have also been found to be associated with loss of hippocampal cells in the CA [26]–[28]. GC-mediated apoptosis of hippocampal neurons is thought to result from increased expression of proapoptotic genes Bad, Puma and Bnip3 [29]–[31]. In both experimental groups, the total number of apoptotic cells in the CA and DG increased in the perinatal period (Fig. 4A,B). This increase in apoptotic cells is most likely the result of a rise in endogenous GC release [17], which plays a major role in the preparation for the transition from intrauterine to extrauterine life [32].

In adulthood we found an increase in the number neurons in the CA, with, however, no increase in volume of the CA. This long term increase in the number of CA neurons has never been described before and it will be interesting to investigate what effect this has on hippocampal function.

**Figure 6:**
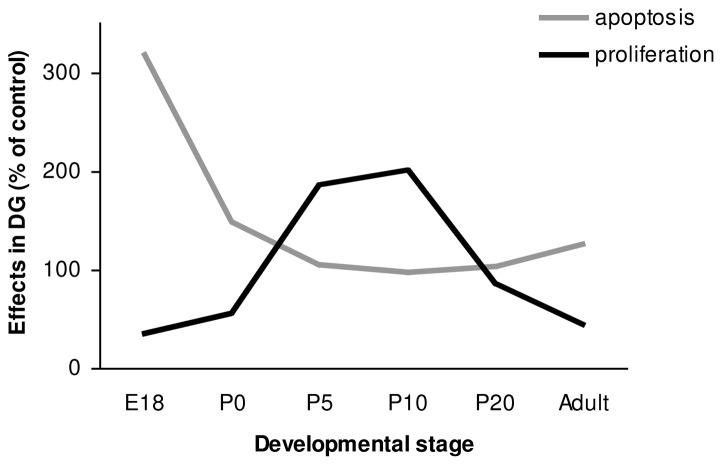
Summary of the effects of antenatal dex treatment on proliferation and apoptosis in the DG during hippocampal development. The results of the dex treated groups are presented as percentage of the control (100%).

In the DG, we found an increase in apoptosis and a decrease in proliferation shortly after dex administration at E18 and at birth. However, these effects were not accompanied by a significant effect on the total number of neurons in the DG and on volume of the DG. The effects of dex on apoptosis in the DG are consistent with the findings of Hassan et al. [6], who described GC-induced cell loss in the DG of adult rats after a single dex administration (60 µg/kg). Surprisingly, in the present study, a single antenatal dex administration had similar effects on apoptosis, despite exposure of the fetus to a much lower dose of dex. The inhibiting effect of GCs on neurogenesis in SGZ of the hippocampus is consistent with the findings of others, who described inhibition of granule cell precursor proliferation within 3 hours of GC injection [33]–[36]. It is thought that GCs decrease proliferation through an N-methyl-D-aspartate (NMDA)-receptor-mediated mechanism [33], [37]. The increase in number of neurons in the DG measured at P10 and P20 and the volume at P10 is likely to be the consequence of the large increase (almost doubled) in the number of proliferating cells found at P5 and P10. The large increase in proliferating cells at P5 appears not to be consistent with the measurement of the total number of neurons in the DG at P5, at which time no difference was found after dex administration. However, this apparent ‘delay’ could be due to the large number of apoptotic cells after dex treatment, which have to be replenished before an increase in total number of neurons can be observed. The increase in proliferation is most likely due to mechanisms involved in neuronal replenishment after apoptosis. The effects of dex on proliferation and apoptosis in the DG during hippocampal development are summarized in figure 6. Many studies have shown a relationship between proliferation and neuron death after ischemia, seizures, brain trauma, and epilepsy [15], [16], [18], [38], [39]. Since apoptosis is often associated with increased neurogenesis, it has been proposed that neuronal progenitors may respond to signals from dying cells by re-entering the cell-cycle. One way in which the processes of apoptosis and neurogenesis could be linked is through the regulation of endogenous neurogenic factors [40]. TGF-β1 is thought to play in important role, since TGF-β receptors are expressed on proliferating cells in the dentate gyrus, and TGF- β1 has been shown to be increased under conditions of cell damage. Upregulation of TGF-β1 after apoptosis may stimulate neuronal progenitors to divide [40]. At P20 and in adulthood, the two groups did not differ in number of proliferative cells, probably because we measured at the end-stage of the repair mechanism after apoptosis (Fig. 6). Finally, at adult stage, when mice are 6 months old, we found a reduced number of proliferating cells in the dex-treated group, while the total number of neurons in the DG was unchanged. It is known that basal neurogenesis declines with advancing age [41], which was also shown in the saline-treated group during hippocampal development. During the life-span the total available number of proliferating cells is more rapidly utilized after apoptosis caused by dex treatment. This long-term effect of dex administration can be due to a restricted amount of proliferation of progenitor cells. Mirescu et al. [42] have shown that the effects of postnatal stress decrease proliferation in adults. Our results indicate that GCs in early life can permanently affect neurogenesis.

Dex treatment induced growth restriction, as measured by body weight, was present at P5 and P10. The decrease in body weight is most likely due to the effects of dex on tissue accretion and catabolism [43]–[45] and to decreased circulating levels of IGF-1 [46]. The effects of dex on birth weight are consistent with one study in human infants born preterm following a single course of antenatal GCs [47]. Nevertheless birth weights from treatment and placebo groups in several trials are similar and provide reasonable evidence that a single course of GCs in humans does not affect fetal growth [48]. Following repeated antenatal GCs courses a weight restriction at birth has been consistently found [49]–[52]. As the dex-treated animals themselves were smaller, a decrease in hippocampal volume might be expected, except that the peripheral vasoconstrictor effects of dexamethasone in early life are well known. For instance, two separate studies have reported that treatment of fetal sheep in late gestation with GCs leads to an increase in femoral vascular resistance [53], [54], leading to asymmetric growth, including a brain sparing effect. In this study we did not measure brain weights, we only measured hippocampal volume. Given the fact that in this study significant effects of dex on body weight were found at different time points (P10 and P20) than the effects of dex on hippocampal volume (at P5 and P10), suggests that hippocampal volume is not only determined by body weight.

Fortunately, the results of antenatal dex treatment on body weight, on the volume of the hippocampus and on apoptosis were transient, suggesting compensation and limited long-term effects. The only long-term effects found, were on the number of neurons in the adult mice CA area and on the number of proliferating cells in the adult hippocampus. Accumulating evidence suggests that adult-born dentate granule cells contribute to learning and memory processes, consistent with computational theories that newborn neurons in the networks are likely to be selected for encoding new information (reviewed in Wang et al. [55]). The impaired neurogenesis that we found after antenatal dex at adult stage suggests an impaired cognitive function. Indeed, previously published data by our group indicates that antenatal dex administration results in impaired spatial learning and memory in adult mice [56]. Follow-up studies after antenatal administration of one course of GCs in human is thus far reassuring, with no adverse effects on the child's physical or mental health and psychomotor development at 1 year, 3 years and 6 years. In one study subtle neurological impairment was present at the age of 6, but physical and physiological development at 12 and 20 years were normal [57]–[60]. Although the general sequence of brain growth and development is similar among species, caution is necessary when extrapolating data from animal models to the human situation. An important consideration is that the maximum velocity of brain growth in mice occurs after parturition, in contrast to humans, where the maximum velocity of brain growth occurs around the time of parturition. The age the pups were exposed to dex treatment was at E15.5, a time point which is comparable to the human situation in the third trimester, as far as hippocampal development is concerned. The dose of dex we used (0.4 mg/kg) was comparable with doses used in other rodent studies published before and almost similar to the human clinical situation were pregnant women receive 4 times a 6 mg intramuscular injection of dex independent of their body weight. Our data suggest that GCs may also in the human affect neurogenesis during adulthood, potentially resulting in cognitive impairment. Such effects might be more pronounced after multiple antenatal courses, a policy that became widespread 10–20 years ago [61], [62]. French et al. [63] found that repeated antenatal courses of corticosteroids (≥3 courses) were associated with increased rates of aggressive/destructive, distractible, and hyperkinetic behavior at both 3 and 6 years of age. From randomized controlled trials in preterm newborns it has become clear that neonatal GC treatment, in which steroid doses are usually higher and treatment is continued for a longer period, leads to abnormal neurological development, cognitive function and cerebral palsy at follow-up [64]–[66]. Further detailed follow-up after antenatal dex treatment in the human is therefore recommended and should focus on hippocampal function, for instance spatial learning.

In conclusion, a clinically relevant dose of antenatal dex resulted in increased apoptosis in both the CA and DG and reduced proliferation in the SGZ of the DG of the fetal hippocampus shortly after dex treatment, followed by enhanced proliferation postnatally. However it also caused permanent deficits in proliferation in the adult hippocampus. The latter observations warrant detailed follow up focused on hippocampal function after antenatal GC treatment.
